# Resuscitation with an AED: putting the data to use

**DOI:** 10.1007/s12471-020-01504-z

**Published:** 2020-10-14

**Authors:** M. A. R. Bak, M. T. Blom, R. W. Koster, M. C. Ploem

**Affiliations:** 1 Section of Medical Ethics, Department of General Practice, Amsterdam UMC, University of Amsterdam, Amsterdam, The Netherlands; 2 ARREST Research Group, Department of Cardiology, Heart Center, Amsterdam UMC, University of Amsterdam, Amsterdam, The Netherlands; 3 Section of Health Law, Department of Social Medicine, Amsterdam UMC, University of Amsterdam, Amsterdam, The Netherlands

**Keywords:** Resuscitation, Automated external defibrillator, Out-of-hospital cardiac arrest, Quality of healthcare, Medical records, Ethics

## Abstract

The increased use of the automated external defibrillator (AED) contributes to the rising survival rate after sudden cardiac arrest in the Netherlands. When used, the AED records the unconscious person’s medical data (heart rhythm and information about cardiopulmonary resuscitation), which may be important for further diagnosis and treatment. In practice, ethical and legal questions arise about what can and should be done with these ‘AED data’. In this article, the authors advocate the development of national guidelines on the handling of AED data. These guidelines should serve two purposes: (1) to safeguard that data are handled carefully in accordance with data protection principles and the rules of medical confidentiality; and (2) to ensure nationwide availability of data for care of patients who survive resuscitation, as well as for quality monitoring of this care and for related scientific research. Given the medical ethical duties of beneficence and fairness, existing (sometimes lifesaving) information about AED use ought to be made available to clinicians and researchers on a structural basis. Creating a national AED data infrastructure, however, requires overcoming practical and organisational barriers. In addition, further legal study is warranted.

## Introduction

Every year, 15,000–16,000 people in the Netherlands are affected by sudden cardiac arrest outside the hospital, with an average survival rate of 23% [1]. This is high compared with the rate in other countries, due in part to the increased deployment of the automated external defibrillator (AED) since its introduction nearly 20 years ago. Nowadays, an AED is connected in more than half of all resuscitations in the Netherlands. For patients with a cardiac arrest and a ‘shockable’ rhythm (ventricular fibrillation or ventricular tachycardia), rapid defibrillation is crucial. In the period of 2006–2014, the increased use of an AED in this group of patients was associated with an increase in the survival rate from 32.4% to 49.5% [2]. Of the surviving patients, 56% received initial defibrillation with an AED [3].

An AED can be deployed by so-called ‘first responders’ (police and fire brigade) after notification by ambulance care dispatch centre. In the Netherlands, AEDs can also be operated by bystanders witnessing the cardiac arrest, since this is no longer an act reserved by law for healthcare professionals [4]. In addition, ‘citizen responders’, who have completed a course on cardiopulmonary resuscitation (CPR), can be paged by the dispatch centre through text message or smartphone app notification as part of a national resuscitation alert system (*HartslagNu*) [5]. These citizen responders often collect a public AED while making their way to the patient.

**Fig. 1:**
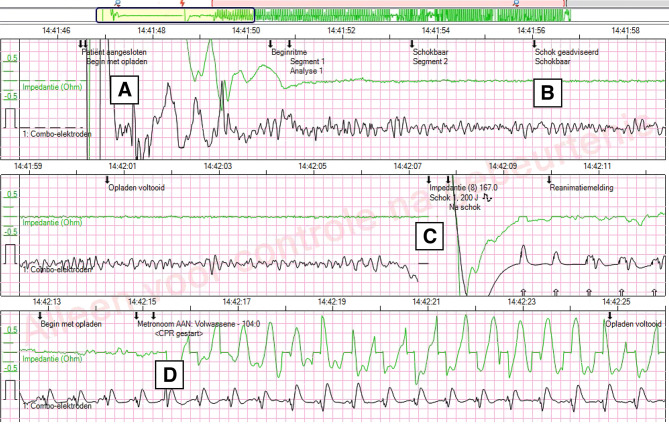
Example of the start of an automated external defibrillator (AED) registration. At timepoint A, the electrodes of the AED are connected and the rhythm analysis starts. At timepoint B (9 s later), the AED has determined there is a ‘shockable’ heart rhythm and charges itself. At timepoint C, the rescuer delivers a shock 7 s after the AED instructed it do so. The heart rhythm has returned to normal immediately. At timepoint D (8 s later), chest compressions are started at a speed of approximately 105 per minute, indicated by the green line. Three important conclusions can be drawn from this registration: (1) the cause of the patient’s unconsciousness was ventricular fibrillation; (2) the first shock was successful; and (3) the (non-medically trained) civilian care provider has acted quickly and adequately in full accordance with the guidelines

When using an AED, the device registers the unconscious person’s heart rhythm, as well as information about the delivery of one or more defibrillation shocks and about the resuscitation procedures, i.e. chest compressions and ventilations (Fig. 1). In this point of view article, we consider the healthcare goals for which data recorded by an AED are of vital importance and the legal framework that applies to such data. We argue that these data are currently underutilised and conclude by giving suggestions on how wider availability of AED data can be achieved.

## Clinical value of AED data

### Good and continuous care

When the ambulance staff take over CPR from first responders or citizens, they check the patient’s heart rhythm. Absence of a shockable rhythm may suggest that earlier defibrillation (by the AED) has been successful—sometimes the patient has even regained consciousness. The AED, however, does not display what happened before the ambulance personnel were on site and obtaining the electrocardiography (ECG) records from the AED requires a complicated read-out procedure. Recent research has shown that in 11–13% of cases of out-of-hospital cardiac arrest, the shockable rhythm can only be seen on the AED’s ECG record, and that adequate transfer of this critical information, first to the ambulance staff and then to the hospital, is not always accomplished [6].

If treating physicians are not aware that the patient was defibrillated with an AED to treat ventricular fibrillation, the cause of the collapse may not be recognised as cardiac arrest. If an AED/ECG record is absent, the patient may not receive the appropriate treatment, such as an implantable cardioverter-defibrillator, and thus remain at risk of a new cardiac arrest. Even when it is known that the AED delivered a shock, using this information as a proxy for ventricular fibrillation can result in treatment errors [7]. Therefore, AED/ECG records should always be analysed to allow for correct clinical decision-making.

Even though these data are necessary to provide good and continuous care of patients who survive a cardiac arrest, to date, systematic collection and storage of data generated by an AED is lacking. The only exception is the Dutch province of North Holland, in which AED/ECG records are routinely read out and stored. However, the primary objective here is not patient care, but scientific research. As such, a valuable registry, known as the AmsteRdam REsuscitation STudies (ARREST; see Box 1), has been created. While it is not the main purpose of the ARREST Research Group, its employees regularly—and often unsolicited—contact the treating physician to give information about the initial rhythm registered by the AED, in order to potentially benefit the patient’s diagnosis and treatment.

#### Box 1: ARREST

The AmsteRdam REsuscitation STudies (ARREST) have been carried out by the Department of Cardiology at the Academic Medical Center in Amsterdam, the Netherlands since 1995, in collaboration with emergency medical services in the Dutch province of North Holland. To gain insight into the causes of and the survival after cardiac arrest, automated external defibrillator (AED) data are read out (on location, by means of special software), and information is collected from patients about hospital treatments, medical history, medication use and hereditary disorders. The use of AED data for ARREST requires the patient’s consent once he or she has recovered sufficiently. Still, it is important to collect the data quickly since the electrocardiogram may otherwise be overwritten.

It is unfortunate that information about defibrillation with an AED only becomes available in the framework of ARREST. This facility does not offer a sustainable solution due to its limited resources and its focus on only a few regions and on scientific research instead of care provision (and evaluation thereof). Only inhabitants of the study regions in North Holland can benefit from the transfer of AED data to their treating physician. This ‘non-use’ of AED data can be questioned in light of important medical ethical values such as beneficence and fairness, i.e. providing good and equal care of all patients experiencing a cardiac arrest [8]. Data collection is challenging and costly, however, with ARREST employees physically traveling to the AED location (when known) to collect the ECG record. Greater availability requires overcoming logistical barriers to the registration of privately owned AEDs and to subsequent data extraction, which is generally not automated nor standardised for different types of defibrillators [9].

### Quality assessment and scientific research

Data collected by AEDs can also be used to evaluate the CPR quality, from an epidemiological perspective or at the individual level, e.g. when providing feedback to first responders. ARREST researchers have experienced that oral feedback (‘You have done well’) is often insufficient and that there is generally a desire for further evaluation through access to data on the resuscitation procedures. Of note, negative feedback (e.g. incorrect rate of chest compressions) is usually given to CPR instructors in general terms rather than to individual responders. In addition to quality monitoring, knowledge of the initial rhythm stored in the AED is required for scientific research that—similar to ARREST—aims to investigate the causes of cardiac arrest or to improve defibrillator performance. Still, researchers should remain mindful of potential individual and group-level harms associated with the use of data from this vulnerable patient group, as we have previously described [10].

## Data protection considerations

In our opinion, it is beyond dispute that ‘AED data’ are important for both care and research. However, making the data available for these purposes requires implementing privacy safeguards to recognise and to support the patient’s autonomy. Moreover, healthcare providers and first responders also have an interest in protection of AED data as they may provide information about the quality of their resuscitation efforts. As such, the processing of AED data (i.e. viewing, using, sharing, et cetera) is regulated by data protection laws, as outlined in Box 2.

### Box 2: Data protection legislation in the Netherlands

*General Data Protection Regulation*

Relevant legislation primarily concerns the General Data Protection Regulation (GDPR) of the European Union, which entered into force in 2018, and the (national) GDPR Implementation Act. From the GDPR it follows that individuals whose data are processed, have rights, such as the right to information and the right to access their data (including information about who has accessed the data). Those who process the automated external defibrillator (AED) data, have duties, such as providing information about data processing, performing a data protection impact assessment (an investigation to assess privacy risks), taking appropriate security measures and reporting data breaches. A complicating factor in the context of resuscitation is the unconscious patient’s inability to exercise his or her own rights at the time and their dependence on a representative. Moreover, the GDPR does not provide privacy protection for deceased individuals (recital 27, preamble GDPR).

*Medical Treatment Act*

If AED data are collected within a professional/patient relationship (for example, when an ambulance employee or general practitioner uses the AED) or are viewed in the context of healthcare evaluation, the Dutch Medical Treatment Act (*Wet op de geneeskundige behandelingsovereenkomst*) applies. From the record and retention obligation (Article 7:454 of the Dutch Civil Code) in the Act it follows that the professional care provider keeps a record of all health data that are deemed necessary for good care, for 20 years or as long as needed. From the medical professional secrecy (Article 7:457 Dutch Civil Code) it follows that, besides the patient, access to data is limited to care providers who need it to perform their duties or evaluate the quality of their actions, and to relatives who represent the interests of their incapacitated family member. The Medical Treatment Act does not directly apply to deceased persons, but offers scope for research with data from deceased patients without their prior consent (Article 7:458 Dutch Civil Code).

Despite the existence of a general legal framework covering the protection of medical data and highlighting the obligation to keep records of relevant data, clear rules regarding the access to AED data are lacking. For instance, are citizen responders allowed to view this personal information for quality monitoring purposes? While their efforts are invaluable to those in need, they are usually not a doctor or other professional bound by medical secrecy as specified in the Medical Treatment Act. The General Data Protection Regulation (GDPR) may not suffice to protect the patient’s data either, as this does not apply to ‘anonymous’ information—this means one cannot uncover the resuscitated person’s identity without disproportionate deployment of manpower and resources. Neither does it apply to information about deceased individuals.

Currently, it is unclear who is responsible for keeping the AED data secure and for performing a data protection impact assessment. There is a lack of oversight, for instance, when owners of an AED buy the software needed to extract these data themselves or when the judiciary takes an interest in the data in relation to a criminal case. How are data protected from these third parties who are not covered by medical secrecy, especially when the patient is deceased and the GDPR does not apply either? What could be the effect if public trust in resuscitation care decreases as a result of missing data protection safeguards?

In addition, it remains difficult to assess under which conditions personal data may be used for scientific research (e.g. consent requirements, see [11]), making some AED owners hesitant towards sharing data with researchers without patient consent. (Obtaining consent prior to collection would be highly impractical given the time-sensitive nature of the data, which will be deleted when the AED is used again.) Both healthcare evaluation and medical research suffer when these data are not sufficiently available because this leads to distorted figures and less representative results.

**Fig. 2:**
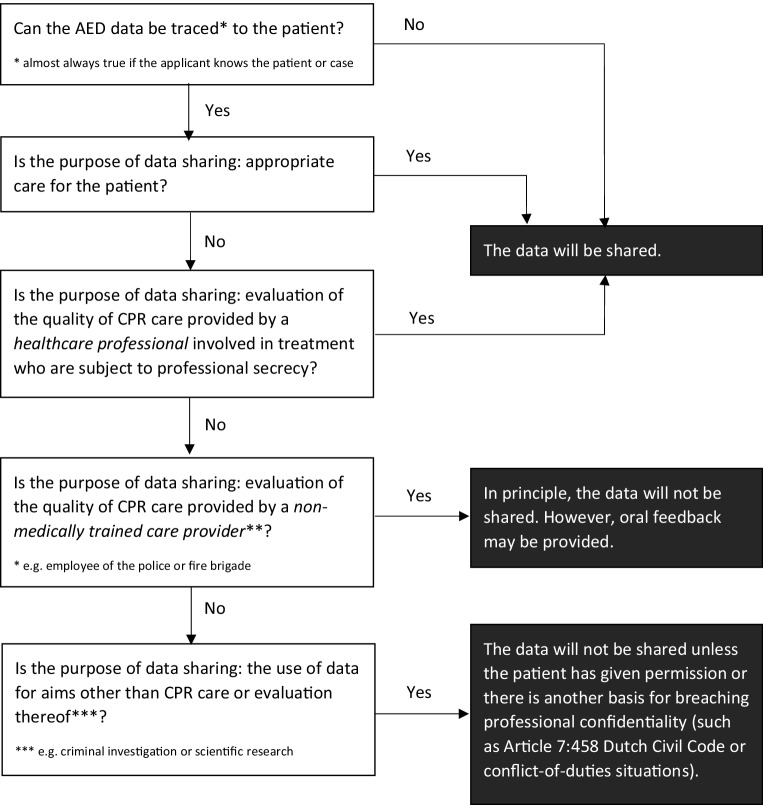
Flowchart of ARREST researchers’ practices of sharing data collected from an automated external defibrillator (AED), such as the AED with ECG, with third parties. *CPR* cardiopulmonary resuscitation

Therefore, there is a need for further legal research on the rules concerning data processing in the context of AED data. In the meantime, we believe that the flowchart used by the ARREST researchers to indicate whether data may be shared, can serve as a good starting point for practice (Fig. 2).

## Conclusion

We conclude that information from the AED is important for patient care, quality assessment and scientific research, and that it ought to be made available for these purposes on a structural basis, given the duties of beneficence and fairness. Collaboration of experts from different fields is essential to overcome logistical, ethical and juridical obstacles and to finally create a national infrastructure for collecting and storing AED data. This AED databank should ideally be linked to a nationwide sudden cardiac arrest registry, the creation of which is being explored by a working group with various stakeholders from the field of emergency medicine, to support quality measurement and research using routinely collected information (such as data from claims [12] and other clinical sources).

This requires, first and foremost, a national policy in which the (sometimes lifesaving) AED/ECG record is integrated into the electronic patient record. Preferably, this is not regulated by a lengthy process of formal legislation, but by a ministerial decision and self-regulation. A national registration system could also include an option for feedback on CPR quality. We believe that such feedback should not only be possible within the legal framework, it is also inextricably connected to the perceived social responsibility of effectively using an AED when someone is in medical need.
